# A Lower Proportion of Regulatory B Cells in Patients with Henoch–Schoenlein Purpura Nephritis

**DOI:** 10.1371/journal.pone.0152368

**Published:** 2016-03-31

**Authors:** Xintong Hu, Jiandong Tai, Zhihui Qu, Songchen Zhao, Li Zhang, Man Li, Xiguang Sun, Yanfang Jiang

**Affiliations:** 1 Genetic Diagnosis Center, The First Hospital of Jilin University, Changchun, China; 2 Key Laboratory of Zoonoses Research, Ministry of Education, The First Hospital of Jilin University, Changchun, China; 3 Jiangsu Co-innovation Center for Prevention and Control of Important Animal Infectious Diseases and Zoonoses, Yangzhou, China; 4 Department of Nephrology, the First Hospital of Jilin University, Changchun, China; Monash University, AUSTRALIA

## Abstract

**Background:**

Henoch—Schoenlein purpura is the one of most common types of systemic vasculitis that involves impaired renal function and Henoch-Schoenlein purpura nephritis (HSPN). The diagnosis of this condition is largely based on immunohistologic detection of immunoglobulin A1-containing immune complex in the glomerular deposits of mesangium. Despite clinical advances, the etiopathogenesis of HSPN is still largely unknown.

**Methods:**

In this study, we enrolled 25 newly diagnosed HSPN patients and 14 healthy controls. Then, fractions of B cell subtypes were determined in venous blood using flow cytometry. The serum interleukin (IL)-10 concentration was determined by enzyme-linked immunosorbent assay.

**Results:**

Compared to those in healthy controls, the numbers of CD38^+^CD19^+^, CD86^+^CD19^+^, CD38^+^CD86^+^CD19^+^, and CD95^+^CD19^+^ B cells per microliter of blood were significantly higher in HSPN patients. In contrast, the numbers of CD5^+^CD19^+^, IL-10^+^CD19^+^, CD5^+^CD1d^+^CD19^+^, and IL-10^+^CD5^+^CD1d^+^CD19^+^ B cells per microliter of blood and the serum IL-10 concentration were significantly lower in HSPN patients. Following treatment, the numbers of CD38^+^CD19^+^ and CD86^+^CD19^+^ B cells per microliter of blood were significantly reduced in HSPN patients. However, the numbers of CD5^+^CD1d^+^CD19^+^, CD5^+^CD1d^+^IL-10^+^CD19^+^, and IL-10^+^CD19^+^ B cells per microliter of blood and the serum IL-10 concentration were significantly increased in HSPN patients following treatment. The estimated glomerular filtration rate (eGFR) was negatively correlated with the number of CD38^+^CD19^+^ B cells but positively correlated with the numbers of IL-10^+^CD19^+^, CD1d^+^CD5^+^CD19^+^, and IL-10^+^CD1d^+^CD5^+^CD19^+^B cells per microliter of blood and the serum IL-10 concentration. The 24-h urinary protein concentration was positively correlated with the number of CD38^+^CD19^+^B cells but negatively correlated with the numbers of IL-10^+^CD19^+^, CD1d^+^CD5^+^CD19^+^, and IL-10^+^CD1d^+^CD5^+^CD19^+^B cells per microliter of blood and the serum IL-10 concentration.

**Conclusion:**

Our results suggest that CD38^+^CD19^+^ and CD1d^+^CD5^+^CD19^+^ B cells (Bregs) contribute to the pathogenesis of HSPN.

## Introduction

Henoch—Schoenlein purpura (HSP) is a systemic vasculitis that affects small vessels. In this condition, patients develop perivascular inflammatory cell infiltrates. It is an immunoglobulin A-related immune complex-mediated disease that adversely affects the skin, joints, and gastrointestinal system, especially the kidney [1,2]. In recent studies, it has been reported that glomerular damage occurs in patients with HSPN, and such damage might be due to the deposition of mesangial Gd-IgA1-containing immune complex, which acts as a potential mediator via mesangial receptors. Subsequently, complement-mediated stimulation of mesangial cells occurs, leading to their proliferation. Moreover, cytokine secretion is also stimulated under such circumstances [3]. However, IgA deposition recurs in some patients even after they undergo renal transplantation [4,5]. In such patients, we detect mild forms of IgA nephropathy (IgAN), because there is deposition of immune complex and nephritic changes [6]. As a result, we usually detect an extrarenal source of antigen and an antibody immune complex in these patients. Furthermore, B cells are divided into different subsets depending on the presence of surface molecules. In the peripheral blood, naive and memory B cells express different amounts of CD27 [7]. This indicates that activated CD27^+^ B cells can establish memory responses [8]. Activated B cells can differentiate into CD38^+^ plasma cells that secrete antibodies [9,10] and cytokines, which enhance the expression of co-stimulation molecules, especially CD86 (which is an established marker of B-cell activation) and CD95 [11,12]. The CD95 receptor is considered to be a key regulator in the activation of germ cell apoptosis [13]. These different subtypes of B cells are known to collaborate and control the responses of the human immune system; however, very little information is available regarding the mechanisms governing the onset of HSPN in patients.

B cells are primary positive regulators that have the ability to produce Ag-specific Ig and multiple cytokines. However, regulatory B cells (Bregs), which are a subset of B cells, have been found to have negative regulatory function [14]. Presently, in murine models with autoimmune disease, scientists have established that Breg subsets have immunosuppressive activity. This includes B cell subsets that express interleukin (IL)-10 and transforming growth factor (TGF)-β, which can facilitate the recruitment and expansion of regulatory T cells (Tregs) [15–22]. In preliminary studies, scientists have proved that Bregs play a critical regulatory role in experimental autoimmune encephalomyelitis (EAE). Moreover, they also suppress intestinal inflammation in murine models [23,24]. In previous studies, we have proved that activated B and T follicular helper (TFH) cells can contribute to the pathogenesis of minimal change disease (MCD) and hepatitis B virus-associated membranous nephropathy (HBV-MN) [25,26]. In addition, we have found that several CD19^+^ B cell subtypes and IL-10^+^ Bregs are differentially expressed in IgA-nephritis patients [27]. Moreover, previous studies have also reported that IL-10–producing B cells are actively involved in regulating Th1 and Th17 responses in a model of collagen-induced arthritis [28]. In these models, B cells that produce IL-10 play a critical role, and a new IL-10^+^ B cell subset was recently characterized by a CD1d^hi^CD5^+^CD19^+^ phenotype [14,29–31]. Presently, very little information is available about the association between Bregs and HSPN.

In this study, we evaluated the numbers (per microliter of blood) of various B cell subtypes and IL-10–producing B cells that were isolated from the peripheral blood of patients with HSPN and healthy controls (HCs). Thus, we investigated the potential association between the differential expression of B cell subtypes before treatment and the dynamic changes in these B cell subtypes after treatment.

## Materials and Methods

### Patients and controls

A total of 25 patients with new onset HSPN (defined by a disease duration of <2 months) were recruited through the inpatient service of the Department of Nephrology of the First Hospital of Jilin University from January 2015 to October 2015. We excluded patients with any of the following conditions: IgA-nephritis, lupus nephritis, other primary glomerulonephritides, neoplastic disease, active peptic ulcer, diabetes mellitus, and viral hepatitis. In the control group, we included 14 HCs who matched the experimental subjects in terms of age, gender and ethnicity. None of the subjects in the control group had a history of any kind of chronic inflammatory disease. All the included patients fulfilled the EULAR/PRINTO/PRES (European League against Rheumatism/Paediatric Rheumatology International Trials Organisation/Paediatric Rheumatology European Society) criteria for HSP, which is defined as purpura plus; the included patients experienced at least one of the following symptoms: 1) abdominal pain; 2) typical leukocytoclastic vasculitis or proliferative glomerulonephritis with IgA deposits, as confirmed by histopathological examination; 3) arthritis or arthralgia; and 4) renal involvement [32]. HSPN patients develop either a nephritic or a nephrotic syndrome. Nephritic syndrome is defined as hematuria with at least one of the following symptoms: renal insufficiency, hypertension, and oliguria. Here, nephrotic syndrome was defined by proteinuria >400 mg/day or hematuria. In some studies, it has been reported that patients with nephrotic syndrome also develop edema and have serum albumin levels <2.5 g/dl [33,34]. All the participants signed a written informed consent form, and this study was approved by the Ethical Committee of the First Hospital of Jilin University. The demographic and clinical characteristics of participants are summarized in Table 1.

**Table 1:** Demographic and clinical characteristics of HSPN patients and HCs.

	HSPN patients (n = 25)	HCs (n = 14)
Age, years	44(16–86)	44(19–74)
Female/male	12/10	8/6
Lymphocytes, 10^9^/L	2.46(0.75–3.45)	1.1 (0.4–1.74)
Serum albumin, g/L	31.3(23–45.8)	42.1(38.7–49)
Serum uric acid, μmol/L	322(225–424)	335(230–440)
Triglycerides, mmol/L	3.02(0.7–4.27)	1.15(0.35–1.63)
Cholesterol, mmol/L	4.14(3.08–5.87)	4.17(2.8–5.95)
Urinary proteins, g/24 h	2.8(0.45–7.2) *	0.05(0–0.15)
Urea nitrogen, mmol/L	4.84(2.93–7.71)	5.05(3.65–6.74)
eGFR, mL/min/1.73m^2^	90.4(16.76–116) *	99 (90–109.14)
Hematuria, n(%)	25(100%)	0(0%)

Data shown are median (range), real number of cases (n/n), and number of cases with percentage [n(%)]. eGFR: estimated glomerular filtration rate. Hematuria: defined as microscopic red blood cells >3 rbc/hpf. Normal values: lymphocytes: 1.10–3.20 (109/L), serum albumin: 40.00–55.00 g/L, serum uric acid: 210–430 μmol/L, triglycerides: 0.28–1.6 mmol/L, cholesterol: 2.6–6.0 mmol/L, urinary protein: <0.2 g/24 h, urea nitrogen: 3.2–7.0 mmol/L, eGFR: 80–120 ml/min.

*P<0.05 vs. HCs.

### Treatment and follow-up

Individual patients were treated orally with glucocorticoid prednisolone (PDN, Tianyao Pharmaceuticals, Tianjin China) in a 1 mg/kg/day dosage for the first 2 months. This dosage of steroids was gradually tapered, and a maintenance dose of 10 mg/day was prescribed to these patients for the next 6 months, or a combination of oral immunosuppressants (tacrolimus capsules, 1 mg/day) to relieve urinary proteinuria. In addition, some patients were at a higher risk of developing a hypercoagulable state, and they were treated with dipyridamole (25 mg/day, Yunpeng Pharmaceutical, Shanxi, China). The patients were followed up for 8–12 weeks. Eight patients had complete records, and the other 17 patients were censored on follow-up. Blood samples for measurement of clinical lab values, B-cell values and IL-10 concentrations were collected before and after the treatment from January 2015 to June 2015.

### Clinical examination

Venous blood samples (10 ml) were obtained from each participant; the blood samples were collected in heparinized tubes. A small volume of blood was used for isolating peripheral blood mononuclear cells (PBMCs) using density-gradient centrifugation (Amersham Biosciences, Little Chalfont, UK). The remaining blood samples were centrifuged for preparing serum samples. We determined the concentrations of serum triglycerides, serum IgA cholesterol, albumin, uric acid, leukocytes and lymphocytes using ADVIA 1650 biochemical analyzer (Bayer, Pittsburg, PA, USA). Moreover, we collected 24-hour urine samples from individual subjects to examine urinary proteinuria and microscopic hematuria. In addition, we calculated the estimated glomerular fitration rate (eGFR) of individual participants using the revised eGFR formula [35].

### Flow cytometric analysis

Human PBMCs were stained at a cell density of 10^6^/tube in duplicates. For the purpose of staining, we used PerCP-anti-CD19, PE-anti-CD38, APC-anti-CD86, or PerCP-anti-CD19, PE-anti-CD27, and APC-anti-CD95 or isotype-matched control IgG (BD Biosciences, San Jose, CA, USA). The staining was performed at room temperature, and the cells were protected from light for 30 minutes. After washing with phosphate-buffered saline (PBS), the cells were characterized using a FACS Calibur flow cytometer (BD Biosciences). Moreover, at least 30000 events per sample were analyzed using FlowJo software v7.6.2 (Ashland, OR, USA) [36].

To analyze the production of intracellular IL-10, we isolated PBMCs. The cells were then plated in 24-well plates (10^6^cells/well) and stimulated with 50 ng/ml phorbol myristate acetate (PMA), 1.0 mg/ml ionomycin and 5.0 mg/ml lipopolysaccharide (LPS; Sigma—Aldrich, St. Louis, MO, USA) in complete RPMI-1640 medium for 2 h at 37°C in an atmosphere of 5% CO_2_. Then, brefeldin A (GolgiPlug; BD Biosciences) was added to each well, and the cells were incubated for another 4 h. Then, the cells were harvested and washed with PBS. Finally, these cells were stained in duplicate for 30 min using APC-anti-CD19, PE-anti-CD1d, and PerCP-anti-CD5 (BD Biosciences). After washing with PBS, the cells were permeabilized using a permeabilization solution (BD Biosciences). Thereafter, they were stained using FITC-anti-IL-10 (eBiosciences, San Diego, CA, USA). Finally, they were analyzed by flow cytometry using the aforementioned procedure. The details of the antibodies used for flow cytometry are provided in S1 Table.

### Enzyme-linked immunosorbent assay (ELISA)

The serum concentration of IL-10 was determined by ELISA using a human IL-10 ELISA kit (Roche Diagnostics, Lewes, UK) according to the manufacturer’s instructions. At 1:4 dilutions, individual serums were briefly subjected to ELISA analysis. Thereafter, the serum concentrations of IL-10 were calculated in individual samples using the standard curve established for recombinant IL-10. The detection limit was 2.5 ng/L.

### Human cell isolation and culture

We isolated B cells from PBMCs of five new onset HSPN patients via flow cytometry and staining with APC-anti-CD19 (BD Biosciences). The B cells were resuspended in D-10 (DMEM supplemented with 10% fetal calf serum, 100 U/mL penicillin, and 100 μg/mL streptomycin). Experiments were carried out in duplicate, and cell numbers were equal among the two groups for each patient: one group of D-10 with 1 μM prednisolone, and the other group without prednisolone. These samples were then distributed in wells of 24-well plates (Corning, Tewksbury, MA, USA) at 0.5–2×10^5^ cells per well. After incubation at 37°C in a humidified atmosphere with 5% CO_2_ for 72 h, all cells in culture wells were harvested and stained with PE-anti-CD1d and PerCP-anti-CD5 (BD Biosciences) as described to determine the percentages of Bregs.

### Statistical analysis

Data are expressed as means, medians, and ranges. The numbers of each type of cells tested were calculated according to the percentages of each type of cells multiplied by the lymphocyte count. The differences between the two groups were analyzed using Mann—Whitney U-test. The differences between pre-treatment and post-treatment patients were analyzed by the Wilcoxon test. The correlations were tested using Spearman’s rank correlation test. Statistical analysis was performed using SPSS 19.0 software (IBM, Armonk, NY, USA). Statistically significant cases were those in which the two-sided P-value <0.5.

## Results

### Patient characteristics

There were no significant differences in the ages and gender ratio of HSPN patients and HCs. There were also no significant differences in the serum albumin concentration, serum uric acid concentration, urea nitrogen concentration, triglycerides, cholesterol, and lymphocyte counts of experimental cases and HCs. However, HSPN patients had significantly higher values of 24-h proteinuria compared to the control group. In addition, HSPN patients had a significantly lower eGFR compared to the HCs (Table 1). The clinical and basic immunological parameters of all individual patients and controls are described in S2 Table.

### Differential expression of B cell subsets

**Fig 1:**
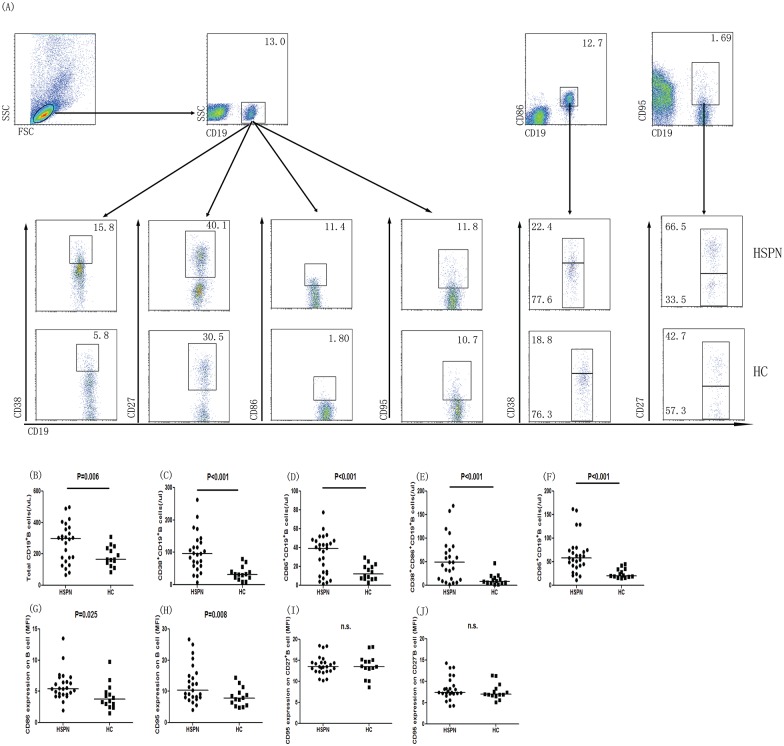
Flow cytometric analysis of the numbers of different subsets of B cells. PBMCs were collected from HSPN patients and healthy controls. Then, they were stained with PerCP-anti-CD19, PE-anti-CD38, APC-anti-CD86, or PerCP-anti-CD19, PE-anti-CD27, and APC-anti-CD95 (Biolegend, San Diego, CA, USA), or isotype-matched control IgG (Beckton Dickinson, San Jose, CA, USA). For further analysis of different subsets of B cells, the cells were gated initially on living lymphocytes and then on CD19^+^ B cells. (A) Flow cytometric analysis results. (B) The numbers of CD19^+^ B cells. (C) The numbers of CD38^+^CD19^+^ plasma cells. (D) The numbers of CD86^+^CD19^+^ B cells. (E) The numbers of CD38^+^CD86^+^CD19^+^ B cells. (F)The numbers of CD95^+^CD19^+^ B cells. Data are expressed as the means for individual subjects included in two separate experiments. (G) Mean fluorescence intensity of CD86 on B cells. (H) Mean fluorescence intensity of CD95 on B cells. (I) Mean fluorescence intensity of CD95 on CD27^+^ B cell subsets. (J) Mean fluorescence intensity of CD95 on CD27^-^ B cell subsets. The horizontal lines represent the median values. Data were analyzed by Mann-Whitney U-test.

In order to elucidate the potential role of different subsets of B cells involved in the pathogenesis of HSPN, we analyzed the numbers of different B cells subsets per microliter of blood of HSPN patients and HCs. The numbers of CD38^+^CD19^+^, CD86^+^CD19^+^, CD38^+^CD86^+^CD19^+^, and CD95^+^CD19^+^B cells per microliter of blood were significantly higher than those in HCs (all P<0.05, Fig 1B–1F). However, there were no differences in the numbers of CD27^+^CD19^+^ and CD27^+^CD95^+^CD19^+^ B cells (data not shown).

### The numbers of IL-10–producing B cells

**Fig 2:**
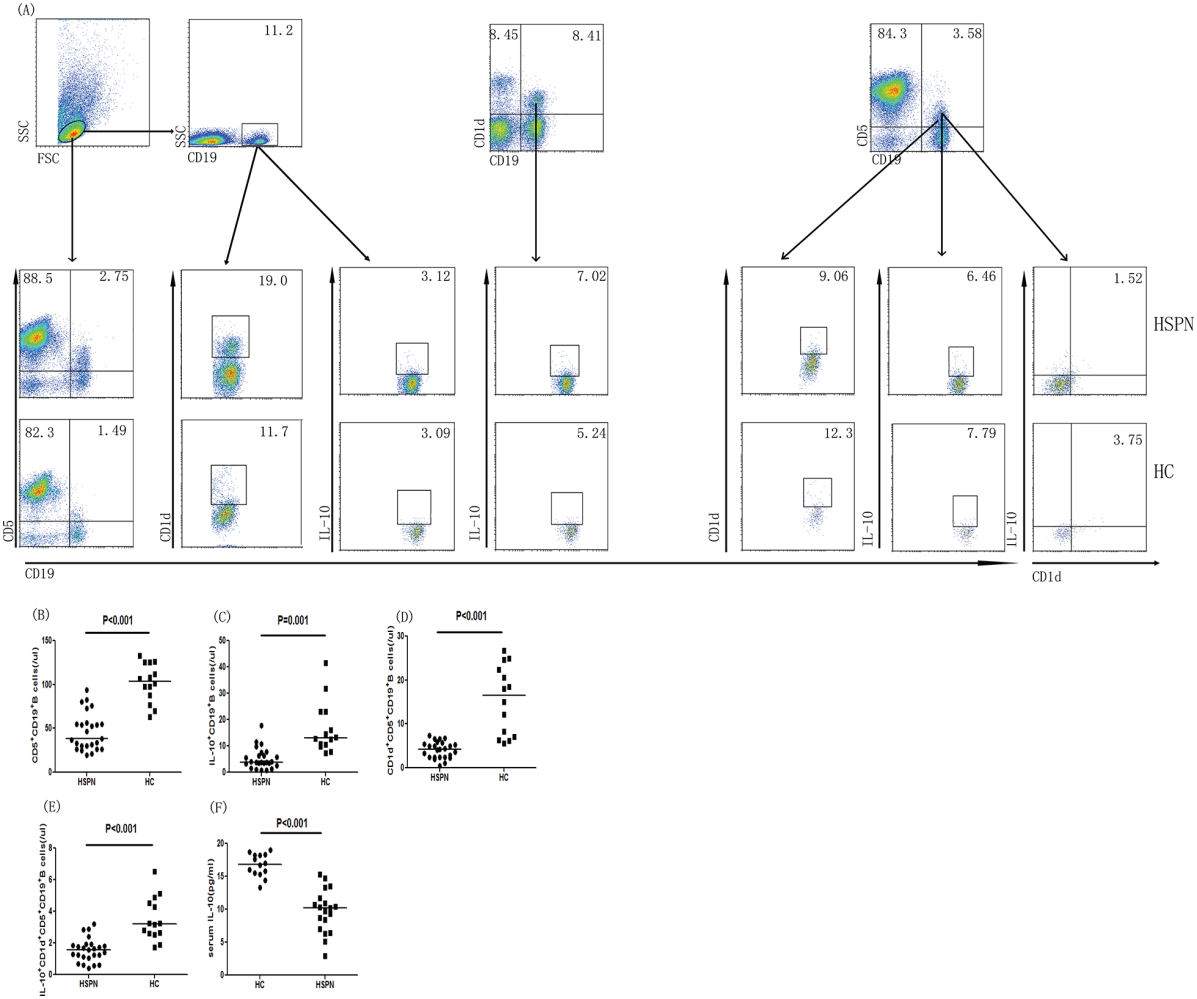
Flow cytometric analysis of Bregs isolated from PBMCs of HSPN patients and healthy controls. The cells were stained with anti-CD19, anti-CD5, anti-CD1d, and intracellular anti-IL-10 or isotype-matched IgG. The cells were characterized by flow cytometric analysis; living lymphocytes were gated initially, followed by gating on CD19^+^ B cells. Subsequently, we analyzed the numbers of CD5^+^CD19^+^, CD1d^+^CD5^+^CD19^+^, and IL-10^+^CD1d^+^CD5^+^CD19^+^ B cells among CD19^+^ B cells, and at least 30,000 events per sample were analyzed. (A) Flow cytometric analysis. (B) The numbers of CD5^+^CD19^+^ B cells. (C) The numbers of IL-10^+^CD19^+^ B cells. (D) The numbers of CD1d^+^CD5^+^CD19^+^ B cells. (E) The numbers of IL-10^+^CD1d^+^CD5^+^CD19^+^ B cells. (F) Serum concentrations of IL-10. Data are expressed as the means or concentrations for individual subjects that participated in two separate experiments. The horizontal lines represent the median values of each group. Data were analyzed by the Mann-Whitney U-test.

We analyzed the numbers of IL-10–producing B cells in patients with HSPN and HCs. The numbers of CD5^+^CD19^+^, CD1d^+^CD19^+^, IL-10^+^CD19^+^, CD1d^+^CD5^+^ CD19^+^ and IL-10^+^CD1d^+^CD5^+^CD19^+^ B cells in HSPN patients were significantly lower than those in HCs (all P<0.05, Fig 2B–2E). Because Bregs play an immunoregulatory role that is primarily mediated via IL-10, we examined the serum concentrations of IL-10 in HSPN patients and HCs. We found that the serum IL-10 concentration was significantly lower in the HSPN patients (P<0.001, Fig 2F). Our raw data can be found in S1 Fig.

### Correlations between B cell subtypes, serum IL-10, and clinical parameters

**Fig 3:**
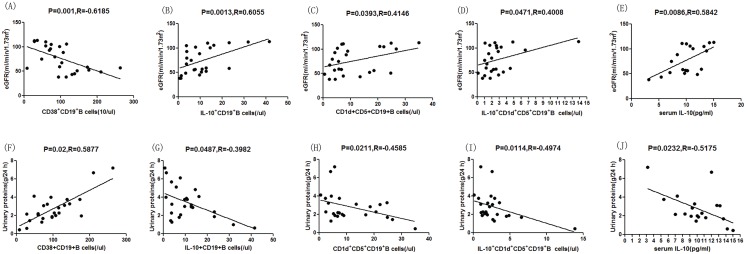
Correlation analysis of clinico-pathological features of HSPN patients, based on the percentages of CD19^+^ B cell subtypes. (A) The eGFR was negatively correlated with the number of CD38^+^CD19^+^ B cells. (B) The eGFR was positively correlated with the numbers of IL-10^+^CD19^+^ B cells, (C) CD1d^+^CD5^+^CD19^+^ B cells (D), and IL-10^+^CD1d^+^CD5^+^CD19^+^ B cells (E) as well as the serum concentration of IL-10. (F) The 24-h urinary protein concentration was positively correlated with the number of CD38^+^CD19^+^ B cells. (G) The 24-h urinary protein concentration was negatively correlated with the numbers of IL-10^+^CD19^+^ B cells, (H) CD1d^+^CD5^+^CD19^+^ B cells, and (I) IL-10^+^CD1d^+^CD5^+^CD19^+^ B cells as well as the (J) serum concentration of IL-10. The potential correlations among the numbers of B cells of different subsets, Bregs, and the values of clinical parameters were analyzed by the Spearman correlation tests.

First, we analyzed the potential relationship between clinical parameters and different subsets of B cells. We found that eGFR was negatively correlated with the number of CD38^+^CD19^+^ B cells (Fig 3A, P = 0.0035, R = -0.6351), while 24-h proteinuria was positively correlated with the number of CD38^+^CD19^+^ B cells per microliter of blood (Fig 3F, R = 0.6684, P = 0.0018). Furthermore, we analyzed the potential relationships of serum IL-10, IL-10–producing B cells, and clinical parameters. In this case, we found that eGFR was positively correlated with the numbers of IL-10^+^CD19^+^ (Fig 3B, P = 0.0003, R = 0.7404), CD1d^+^CD5^+^CD19^+^ (Fig 3C, P = 0.0397, R = 0.4754), and IL-10^+^CD1d^+^CD5^+^CD19^+^ B cells (Fig 3D, P = 0.0477, R = 0.4596) per microliter of blood and the IL-10 serum concentration (Fig 3E, P = 0.0086, R = 0.5842). However, the 24-h proteinuria was negatively correlated with the numbers of IL-10^+^CD19^+^ (Fig 3G, P = 0.0077, R = –0.5912), CD1d^+^CD5^+^CD19^+^ (Fig 3H, P = 0.0154, R = –0.5467), and IL-10^+^CD1d^+^CD5^+^CD19^+^ B cells (Fig 3I, P = 0.0021, R = –0.6591) per microliter of blood and serum concentration of IL-10 (Fig 3J, P = 0.0232, R = –0.5175). In addition, there was no significant effect of age on the numbers of CD1d^+^CD5^+^CD19^+^ Bregs or IL-10^+^CD1d^+^CD5^+^CD19^+^ B cells (data not shown).

### Correlations between CD19+B cell subtypes and the proportion of IL-10–producing B cells

**Fig 4:**
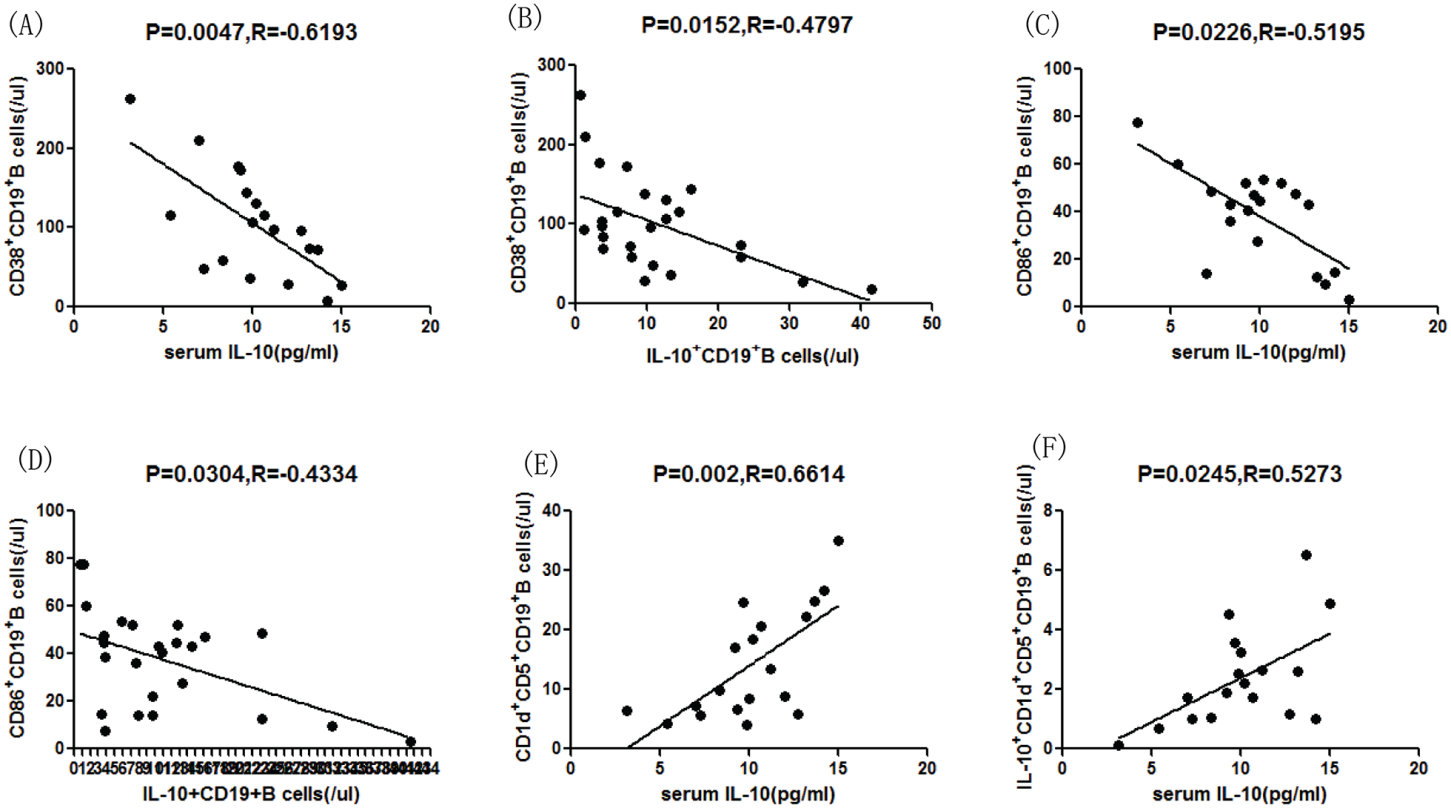
Correlations among different subsets of B cells and the serum concentration of IL-10 in HSPN patients. Potential correlations among the numbers of B cells of different subsets and the serum concentration of IL-10 were analyzed by the Spearman correlation tests. Data are expressed as the means or concentrations for individual subjects that participated in two separate experiments. (A-B) The number of CD38^+^CD19^+^ B cells was negatively correlated with the serum IL-10 level and the percentage of IL-10^+^CD19^+^ B cells. (C-D) The number of CD86^+^CD19^+^ B cells was negatively correlated with the serum IL-10 level and the number of IL-10^+^CD19^+^ B cells. (E-F) The serum IL-10 level was positively correlated with the numbers of CD1d^+^CD5^+^CD19^+^ and IL-10^+^CD1d^+^CD5^+^CD19^+^ B cells.

The fraction of CD38^+^CD19^+^ B cells was negatively correlated with the serum IL-10 concentration (Fig 4A, P = 0.0047, R = –0.6193) and the number of IL-10^+^CD19^+^ B cells per microliter of blood (Fig 4B, P = 0.0244, R = –0.514). In addition, the number of CD86^+^CD19^+^ B cells was negatively correlated with the serum IL-10 concentration (Fig 4C, P = 0.0226, R = –0.5195) and the number of IL-10^+^CD19^+^ B cells (Fig 4D, P = 0.0145, R = –0.5509). In contrast, the numbers of CD1d^+^CD5^+^CD19^+^ (Fig 4E, P = 0.002, R = 0.6614) and IL-10^+^CD1d^+^CD5^+^CD19^+^ B cells (Fig 4F, P = 0.0245, R = 0.5273) per microliter of blood were positively correlated with the serum IL-10 concentration.

### Clinical parameters, different subsets of B cells, and serum IL-10 concentration in HSPN patients following treatment

**Fig 5:**
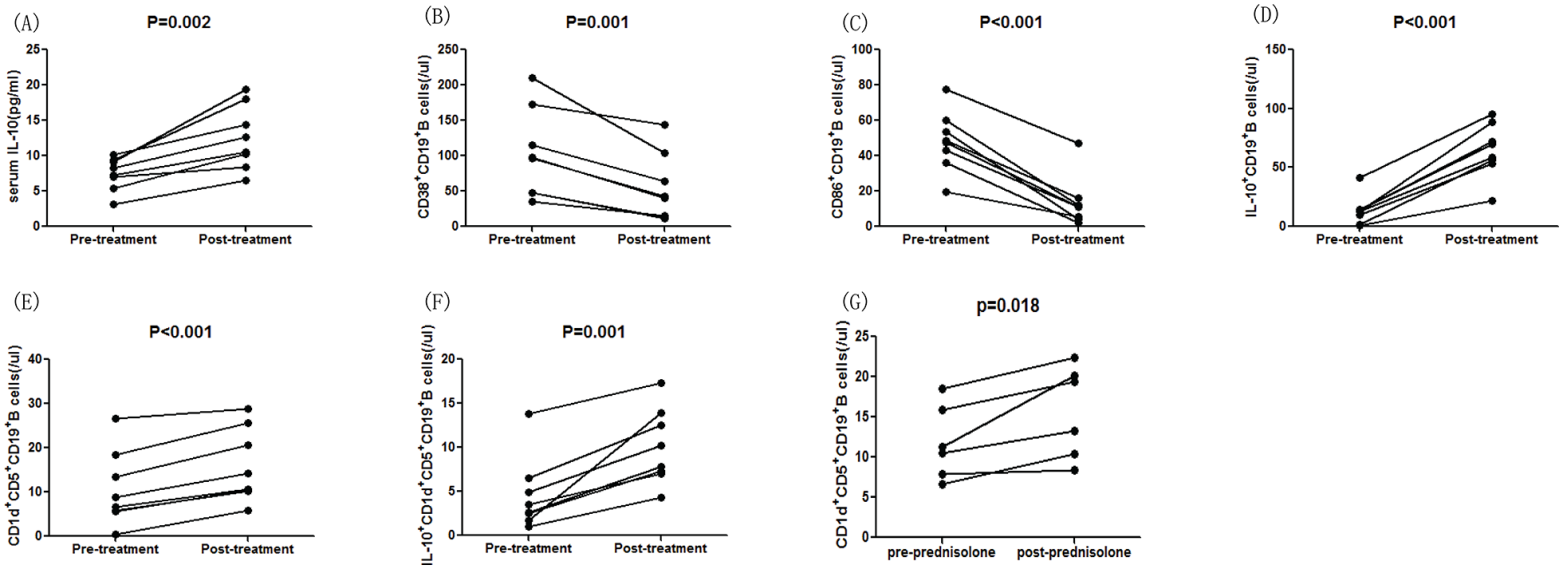
The change in the frequency of B cell subtypes and the serum concentration of IL-10 in HSPN patients following treatment. Differences in patients pre- and post-treatment were analyzed by the Wilcoxon test. Data are expressed as the means or concentrations for individual subjects who participated in two separate experiments. (A) Serum level IL-10 in individual patients’ pre- and post-treatment. (B-F) The numbers of CD38^+^CD19^+^, CD86^+^CD19^+^, IL-10^+^CD19^+^, CD5^+^CD1d^+^CD19^+^, and CD5^+^CD1d^+^IL-10^+^CD19^+^ B cells of individual patients in the pre- and post-treatment stages.

**Fig 6:**
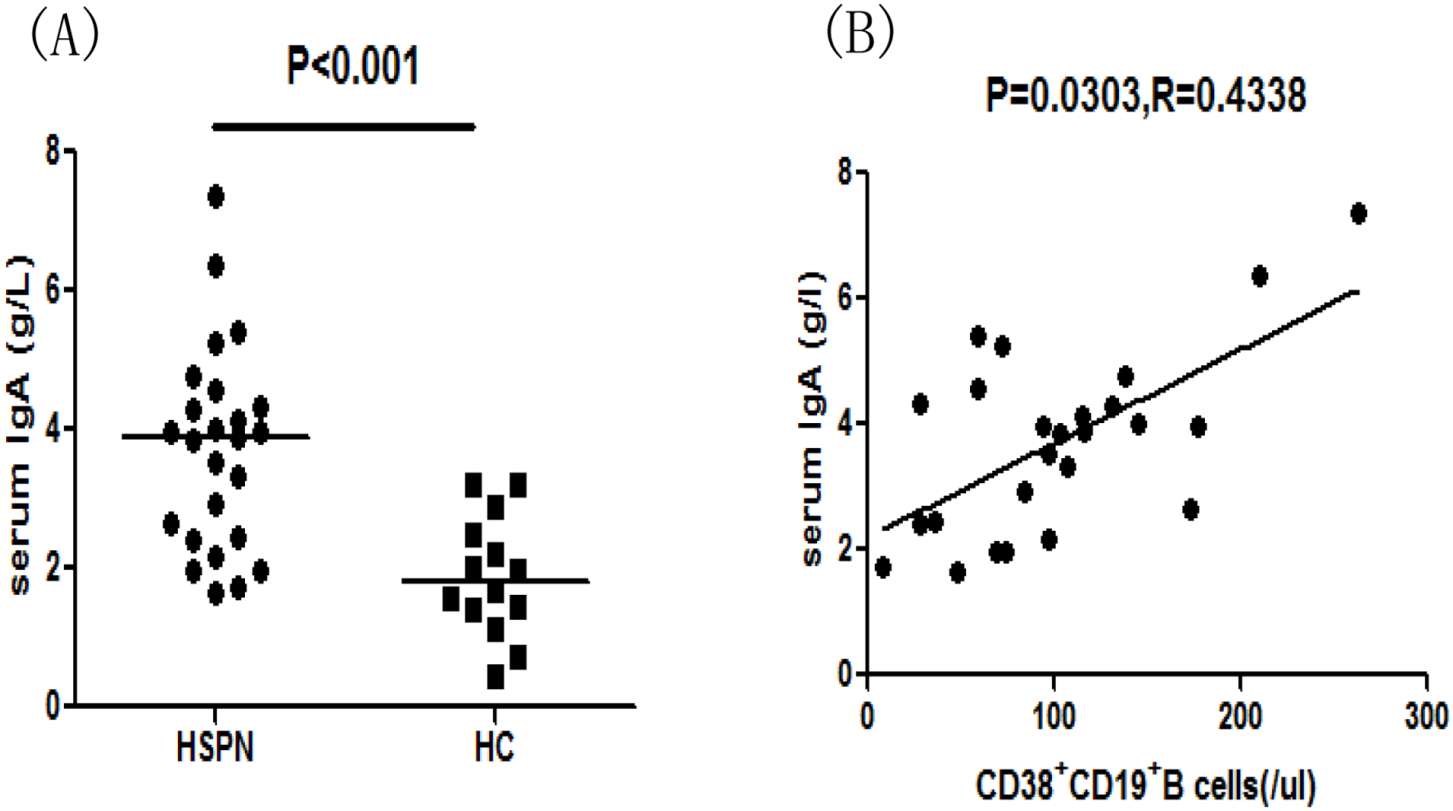
Correlation between the number of CD38^+^CD19^+^ B cells and the serum IgA concentration in HSPN patients. (A) Serum IgA concentrations in HSPN patients and healthy controls. Data are expressed as the means or concentrations for individual patients who participated in two separate experiments. The horizontal lines indicate the median values for each group. (B) The serum IgA concentration was positively correlated with the number of CD38^+^CD19^+^ B cells. Analyzed by Spearman correlation tests.

In order to better understand the function of different subsets of B cells and serum IL-10 in the progression of HSPN, we assessed the values of clinical parameters, the numbers of B cells of different subsets, and the serum IL-10 concentrations in eight patients who reported for follow-up sessions within 8–12 weeks. We found that 24-h proteinuria had decreased significantly in these patients, whereas eGFR had increased (Table 2). In addition, the serum level of IL-10 had significantly increased as compared to the pre-treatment levels (Fig 5A, P = 0.002). Similarly, the numbers of IL-10^+^CD19^+^ (Fig 5D, P<0.001), CD1d^+^CD5^+^CD19^+^ (Fig 5E, P<0.001), and IL-10^+^CD1d^+^CD5^+^CD19^+^ B cells (Fig 5F, P = 0.001) had increased compared to pre-treatment values. Conversely, the numbers of CD38^+^CD19^+^ (Fig 5B, P = 0.001) and CD86^+^CD19^+^ B cells (Fig 5C, P<0.001) had decreased compared to the pre-treatment values. Then, we analyzed the correlation between the number of CD38^+^CD19^+^ B cells and the serum IgA concentration in HSPN patients and found that the serum IgA concentration was positively correlated with the number of CD38^+^CD19^+^ B cells (Fig 6B, P = 0.0303, R = 0.4338). Moreover, the number of Bregs post-treatment was significantly higher than that pre-treatment (Fig 5G, P = 0.018) based on in vitro culture.

**Table 2:** The effect of treatment on the clinical parameters of HSPN patients during the follow-up period.

	Before treatment	After treatment
Age, years	43(18–74)	43(18–74)
Female/male	3/5	3/5
Lymphocytes, 10^9^/L	2.65(1.58–2.93)	2.63(1.27–2.79)
Serum albumin, g/L	30.2(25–39.5)	33.2(25.5–40.7)
Serum uric acid, μmol/L	321(281–398)	312(267–380)
Triglycerides, mmol/L	3.02(1.05–4.27)	2.98(1.34–3.92)
Cholesterol, mmol/L	4.34(3.16–5.87)	4.02(2.94–5.35)
Urinary proteins, g/24 h	3.6(2.38–7.2)	2.55(1–5.62) *
Urea nitrogen, mmol/L	5.31(3.36–7.71)	4.65(3.22–6.98)
eGFR, mL/min/1.73m^2^	88(16.76–102)	91.2(26.89–108.6)*
Hematuria, n(%)	8(100%)	2(25%)

Data shown are median (range), real number of cases (n/n), and number of cases with percentage [n(%)]. eGFR: estimated glomerular filtration rate. Hematuria: defined as microscopic red blood cells >3 rbc/hpf. Normal values: lymphocytes: 1.10–3.20 (109/L), serum albumin: 40.00–55.00 g/L, serum uric acid: 210–430 μmol/L, triglycerides: 0.28–1.6 mmol/L, cholesterol: 2.6–6.0 mmol/L, urinary proteins: <0.2 g/24 h, urea nitrogen: 3.2–7.0 mmol/L, eGFR: 80–120 ml/min.

*P<0.05 vs. the values before treatment.

In summary, the treatment dramatically improved proteinuria in patients. This finding was accompanied by a reduction in the number of CD38^+^D19^+^ B cells. However, there were increases in the numbers of IL-10^+^CD19^+^, CD1d^+^CD5^+^CD19^+^, and IL-10^+^CD1d^+^CD5^+^CD19^+^ B cells and the serum IL-10 concentration in HSPN patients following treatment.

## Discussion

Previous studies have failed to clarify the pathogenesis of HSPN. However, they have proved that HSPN is mainly associated with glomerular deposits of an immune complex containing IgA. These deposits are primarily found in mesangium. Moreover, the deposition of IgA occurs again in some patients who have previously undergone renal transplantation [3]. In our study, we have characterized the roles of different subsets of peripheral blood B cells in HSPN patients and HCs. In our study, the different B cell subsets were defined as CD38^+^CD19^+^, CD86^+^CD19^+^, CD38^+^CD86^+^CD19^+^, CD27^+^CD19^+^ (activated B cells), CD27^-^CD19^+^ (naive B cells), CD95^+^CD19^+^, and CD27^+^CD95^+^CD19^+^B cells. Thereafter, we analyzed the numbers of these cells per microliter of blood. We found that compared to those in HCs, the numbers of CD38^+^CD19^+^, CD86^+^CD19^+^, CD38^+^CD86^+^CD19^+^, and CD95^+^CD19^+^B cells were significantly higher in patients with HSPN. However, there were no significant differences in the numbers of CD27^+^CD19^+^ and CD27^+^CD95^+^CD19^+^ B cells between the experimental and control groups. The high expression of CD86, which activates CD4^+^ T cells, indicates the activation of B cells [12]. Activated B cells can also secrete cytokines and express co-stimulatory molecules, such as CD86 and CD95 [11]. Furthermore, it has been proposed that CD95 acts as a key regulator in the activation of germ cell apoptosis [13]. This feedback regulation can maintain the homeostasis of B cells in HSPN patients. We also analyzed the associations between clinical parameters and the numbers of B cells of different subtypes per microliter of blood. We found that 24-h proteinuria is positively correlated with the number of CD38^+^CD19^+^ B cells, whereas eGFR is negatively correlated with the number of CD38^+^CD19^+^ B cells. Furthermore, the numbers of CD38^+^CD19^+^ and CD86^+^CD19^+^ (activated) B cells were significantly reduced after treatment. Consequently, the number of circulating CD86^+^CD19^+^ B cells, specifically CD38^+^CD19^+^ B cells, may be associated with the pathogenesis of HSPN.

In this experimental study, we also analyzed a new subset of B cells, which are termed Bregs; these cells are characterized by the CD1d^hi^CD5^+^CD19^+^ phenotype [14,29–31]. We were surprised to find that compared to those in HCs, the numbers of CD5^+^CD19^+^, CD1d^+^CD19^+^, IL-10^+^CD19^+^, CD1d^+^CD5^+^CD19^+^, and IL-10^+^CD1d^+^CD5^+^CD19^+^ B cells were significantly lower in HSPN patients. Furthermore, 24-h proteinuria was negatively correlated with the serum IL-10 concentration and the numbers of IL-10^+^CD19^+^, CD1d^+^CD5^+^CD19^+^, and IL-10^+^CD1d^+^CD5^+^CD19^+^ B cells. In addition, the eGFR was positively correlated with the serum IL-10 concentration and the numbers of IL-10^+^CD19^+^, CD1d^+^CD5^+^CD19^+^, and IL-10^+^CD1d^+^CD5^+^CD19^+^ B cells. Interestingly, serum IL-10 levels and the numbers of IL-10^+^CD19^+^, CD1d^+^CD5^+^CD19^+^, and IL-10^+^CD1d^+^CD5^+^CD19^+^ B cells were significantly greater in patients following treatment. Based on these results, we speculate that Bregs and the serum IL-10 concentration play pivotal roles in the pathophysiology of HSPN.

Previous studies have also shown that Bregs play a critical role in the regulation of experimental autoimmune encephalomyelitis (EAE) and in the suppression of intestinal inflammation in murine models via IL-10, which has strong anti-inflammatory activity and inhibitory activity on the immune system [23,24]. Therefore, we analyzed the correlations between CD19^+^ B cell subtypes and the IL-10–producing B cells. We have found that the number of CD38^+^CD19^+^B cells was negatively correlated with the serum IL-10 concentration and the number of IL-10^+^CD19^+^ B cells per microliter of blood. Conversely, the numbers of CD1d^+^CD5^+^CD19^+^and IL-10^+^CD1d^+^CD5^+^CD19^+^ B cells per microliter of blood were positively correlated with the serum IL-10 concentration. These results support the above assumptions, which indicate that a decrease in the number of Bregs can in turn reduce the serum level of IL-10. Consequently, there is a reduction in the immunosuppressive effect, leading to a high number of CD38^+^CD19^+^ B cells in patients with HSPN. An enhanced immune response may stimulate the expression of antigen, and as a result, the deposition of antibody immune complex may be favored in the mesangium, promoting the pathogenesis of HSPN.

In conclusion, our data indicate that CD38^+^CD19^+^ B cells and Bregs may participate in the pathogenesis of HSPN. Because HSPN is mainly associated with glomerular deposits of an immune complex containing IgA, we also analyzed the serum level of IgA in patients with HSPN and found that the serum IgA concentration was positively correlated with the number of CD38^+^CD19^+^ B cells. In addition, a previous study has indicated that 24-h urinary protein can predict the pathological classification of HSPN [37]. Given that HSPN is a serious pathological condition arising from Henoch—Schoenlein purpura, we analyzed the numbers of neutrophils from the patients with HSPN to explore whether our findings represent only a general inflammatory response. We found that the number of Bregs has no correlation with the number of neutrophils (data not shown), suggesting that our findings do not represent a general inflammatory response. Although previous studies have shown that the levels of CD5^+^ B cells and IL-10^+^ B cells are decreased in antineutrophil cytoplasmic antibody-associated vasculitis [38–41] and the frequency of IL-10–producing B cells is higher in hepatitis B virus-associated membranous nephropathy [42], our study is the first to indicate that CD38^+^CD19^+^ B cells and Bregs may participate in the pathogenesis of HSPN.

Our findings suggest that CD38^+^CD19^+^ B cells and Bregs may contribute to the pathogenesis of HSPN, and we also propose that CD38^+^CD19^+^ B cells and Bregs can serve as biomarkers in the evaluation of the severity of disease. However, our research design has some limitations, including the very small sample size. Moreover, a longitudinal follow-up was not observed among the included subjects and we did not carry out any single time-point measurements. Furthermore, our findings regarding CD38^+^ B cells could result from correlations with either transitional B cells or plasma blasts [43]. Also, the CD27^-^CD19^+^ B cells are a mixture of IgM^+^ naive B cells and IgM^-^IgD^-^ memory B cells (IgA, IgG, or IgE) [44,45]. Thus, further dissection of the actual association with either of these subsets is needed. Nonetheless, our data provide novel insights into the unknown pathophysiology and mechanisms of HSPN. Further studies should be conducted to explore the roles of different subsets of B cells in the pathogenesis of HSPN and to gain an understanding of the mechanisms of regulation and activation of Bregs. Such studies are likely to identify a new target that can be used for medical intervention in HSPN patients.

## Supporting Information

S1 Fig The raw data of Bregs in 25 HSPN patients. (ZIP)

S1 Table Details of antibodies used for flow cytometry. (ZIP)

S2 Table Clinical and basic immunological parameters. (ZIP)
