# Supplementary figures and images for: A Lower Proportion of Regulatory B Cells in Patients with Henoch–Schoenlein Purpura Nephritis
Source: PLoS One. 2016 Mar 31;11(3):e0152368. doi: 10.1371/journal.pone.0152368 (PMC4816555; doi:10.1371/journal.pone.0152368)

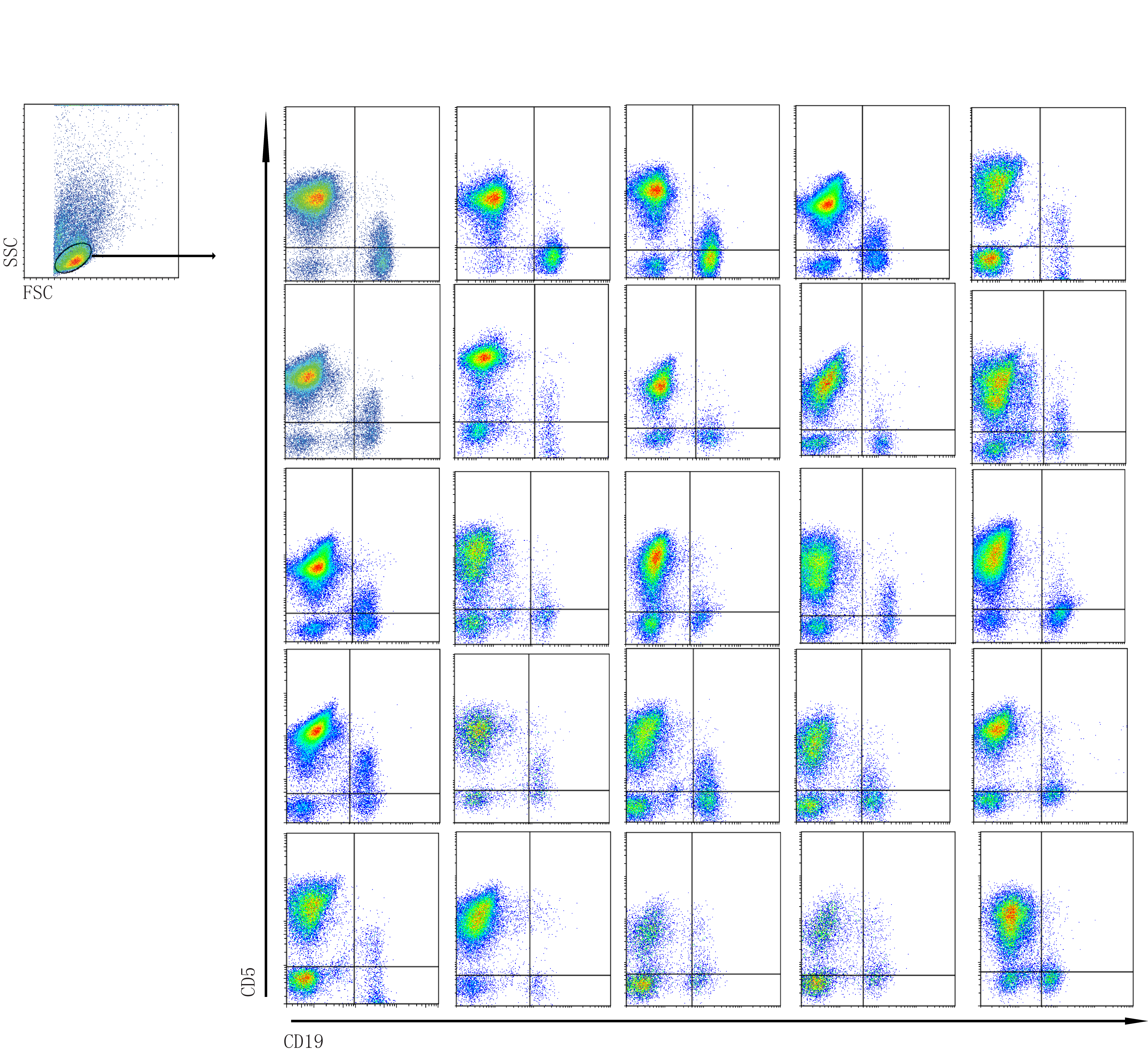

Supplement: S1 Fig — (ZIP) [file pone.0152368.s001.zip › S1 Fig.tif]
