# Supplementary material for: A Lower Proportion of Regulatory B Cells in Patients with Henoch–Schoenlein Purpura Nephritis
Source: PLoS One. 2016 Mar 31;11(3):e0152368. doi: 10.1371/journal.pone.0152368 (PMC4816555; doi:10.1371/journal.pone.0152368)
Supplement: S1 Table — (ZIP) [file pone.0152368.s002.zip › S1 Table.docx]

**S1 Table. Details of antibodies used for flow cytometry.**

|  | Fluorochrome | Clone, Isotype | Company |
| --- | --- | --- | --- |
| anti-CD19 | PerCP | HIB19,  Mouse IgG1, κ | BD Biosciences |
| anti-CD38 | PE | HIT2,  Mouse IgG1, κ | BD Biosciences |
| anti-CD86 | APC | 2331 (FUN-1),  Mouse IgG1, κ | BD Biosciences |
| anti-CD27 | PE | M-T271,  Mouse IgG1, κ | BD Biosciences |
| anti-CD95 | APC | DX2,  Mouse IgG1, κ | BD Biosciences |
| anti-CD19 | APC | HIB19,  Mouse IgG1, κ | BD Biosciences |
| anti-CD1d | PE | CD1d42(42.1),  Mouse IgG1, κ | BD Biosciences |
| anti-CD5 | PerCP | L17F12,  Mouse IgG2a, κ | BD Biosciences |
| anti-IL-10 | FITC | JES3-9D7,  Mouse IgG1, κ | BD Biosciences |
