# Supplementary material for: A Lower Proportion of Regulatory B Cells in Patients with Henoch–Schoenlein Purpura Nephritis
Source: PLoS One. 2016 Mar 31;11(3):e0152368. doi: 10.1371/journal.pone.0152368 (PMC4816555; doi:10.1371/journal.pone.0152368)
Supplement: S2 Table — (ZIP) [file pone.0152368.s003.zip › S2 Table.docx]

**S2 Table. Clinical and basic immunological parameters.**

|  | Normal ranges | HSPN patients (25) | HCs (n=14) |
| --- | --- | --- | --- |
| Lymphocytes,109/L | 1.10-3.20 | 2.46(0.75-3.45) | 1.1 (0.4-1.74) |
| Serum albumin, g/L | 40.00-55.00 | 31.3(23-45.8) | 42.1(38.7-49) |
| Serum uric acid, μmol/L | 210-430 | 322(225-424) | 335(230-440) |
| Triglycerides, mmol/L | 0.28-1.6 | 3.02(0.7-4.27) | 1.15(0.35-1.63) |
| Cholesterol, mmol/L | 2.6-6.0 | 4.14(3.08-5.87) | 4.17(2.8-5.95) |
| Urinary proteins, g/24 h | <0.2 | 2.8(0.45-7.2) * | 0.05(0-0.15) |
| Urea nitrogen, mmol/L | 3.2-7.0 | 4.84(2.93-7.71) | 5.05(3.65-6.74) |
| eGFR, mL/min/1.73m2 | 80-120 | 90.4(16.76-116) * | 99 (90 -109.14) |
| Neutrophils, 109/L | 1.8-6.3 | 2.6(1.1-8.3) | 2.2(1.5-4.48) |
| Serum IgA, g/L | 0.7-4.0 | 3.85(1.6-7.4) * | 2.0(0.2-3.2) |

***P<0.05 vs. HCs.**
